# Limited evidence for the usage of renin–angiotensin–aldosterone pathway blockers to prevent arthrofibrosis after total knee arthroplasty. A systematic review of clinical evidence

**DOI:** 10.1002/jeo2.70089

**Published:** 2024-12-11

**Authors:** Giuseppe Anzillotti, Andreas H. Gomoll, Pietro Conte, Alberto Bulgarelli, Paolo Queirazza, Maurilio Marcacci, Elizaveta Kon, Berardo Di Matteo

**Affiliations:** ^1^ IRCCS Humanitas Research Hospital Rozzano Milan Italy; ^2^ Department of Biomedical Sciences Humanitas University Pieve Emanuele Milan Italy; ^3^ Department of Sports Medicine Hospital for Special Surgery New York New York USA

**Keywords:** aldosterone, angiotensin, arthrofibrosis, knee, losartan, RAAS inhibitors, stiffness, TKA, total knee arthroplasty

## Abstract

**Purpose:**

Despite advances in surgical techniques and rehabilitation protocols, arthrofibrosis following total knee arthroplasty (TKA) still has poor outcomes. In the last decade, attention has been focused on the pathogenesis and cascade of events leading to the development of fibrosis. Currently, one of the most promising approaches consists in the indirect antagonisation of transforming growth factor beta 1 (*TGF‐beta 1*) through the downregulation of the renin–angiotensin–aldosterone system (RAAS). This systematic review aims to analyse the available evidence regarding the use of angiotensin receptor blockers (ARBs)/angiotensin‐converting‐enzyme inhibitors (ACEi) in order to prevent post‐operative knee arthrofibrosis following TKA.

**Methods:**

Extensive research on the PubMed, Cochrane, and Google Scholar databases was performed on 8 July 2024, using keywords related to ARBs, ACE inhibitors and arthrofibrosis. Inclusion criteria included: (1) clinical trials of any level of evidence; (2) written in English; (3) studies conducted on humans; and (4) evaluating the antifibrotic effects of ACE inhibitors or ARBs administered for TKA surgeries. Exclusion criteria were articles written in other languages; preclinical studies; expert opinions; reviews and trials evaluating the effects of ACEi/ARBs not related to their antifibrotic effect after TKA.

**Results:**

A total of six studies met the inclusion criteria and were analysed. All studies were retrospective and involved a total of 158,310 patients. Time of administration varied among the studies as well as the dosage, which fell within the range for cardiological use. Four out of six studies focused exclusively on losartan. Three studies reported a clear, significant correlation between the use of ARBs and/or ACEi and a reduced likelihood of developing arthrofibrosis.

**Conclusions:**

The RAAS antagonism could have potential for stiffness prevention after TKA. However, given the side effects and the limited evidence available, the use of ACEi/sartans for the sole purpose of avoiding arthrofibrosis after TKA is not currently recommended.

**Level of Evidence:**

Level III.

**Abbreviations**
ACEi: angiotensin‐converting enzyme inhibitors
AKI: acute kidney injury
ARB: angiotensin receptor blocker
COX 2: cyclooxygenase 2
ED: emergency department
IL‐13: interleukin‐13
IL‐133: interleukin‐133
IL‐1b: interleukin‐1 beta
IL‐6: interleukin‐6
JA: joint arthroplasty
LOA: arthroscopic lysis of adhesions
MACCE: major adverse cardiac, and cerebrovascular event
MDSC: muscle derived stem cell
MUA: manipulation under anaesthesia
PRISMA: Preferred Reporting Items for Systematic Review and Meta‐Analysis
RAA: renin angiotensin antagonists
RAAS: renin–angiotensin–aldosterone system
ROM: range of motion
TGF‐beta 1: transforming growth factor beta 1
TKA: total knee arthroplasty

## INTRODUCTION

Arthrofibrosis or, more appropriately defined, acquired idiopathic stiffness is a condition characterised by an exaggerated intra‐articular fibrotic response of the joint following a surgical insult, in which the scarring tissue prevents a normal range of motion (ROM) and significantly impacts the quality of life of those affected [15, 22]. It is estimated to develop annually in up to 85,000 knees following surgery, with up to 3%–10% occurring after total knee arthroplasty (TKA) [1, 40, 48].

Despite advances in surgical techniques, post‐operative protocols and physiotherapy approaches, the incidence of this complication persists, with associated poor outcomes, reduced joint function, pain and patient dissatisfaction [51].

The first therapeutic approach to this complication is aggressive physiotherapy, but this often proves to be insufficient, thus leading to more invasive procedures such as manipulation under anaesthesia (MUA) or arthroscopic/open arthrolysis [19].

The current state of the art does not suggest an effective solution to this problem [35]. Arthrofibrosis stands as a current and demanding challenge in the mission to improve the outcomes of orthopaedic surgical practice, especially in light of the increasing number of knee surgeries [13, 31].

In the last decade, attention has been focused on the pathogenesis and cascade of events leading to the development of fibrosis, identifying new molecules and pathways. In detail, the surgical insult is able to activate immune cells, which secrete cytokines responsible for the differentiation of fibroblasts into myofibroblasts. Under normal circumstances, the quantity of myofibroblasts gradually declines; however, in certain individuals, the *TGF‐β1*‐induced inflammatory chain reaction further stimulates myofibroblasts in the periarticular tissue to enhance collagen deposition, ultimately leading to stiffness [23, 55]. Hence, the key role of *TGF‐beta 1* in the dysregulation of the inflammatory response has raised new questions and increased interest on the possibility of preventive action [32, 38].

**Figure 1:**
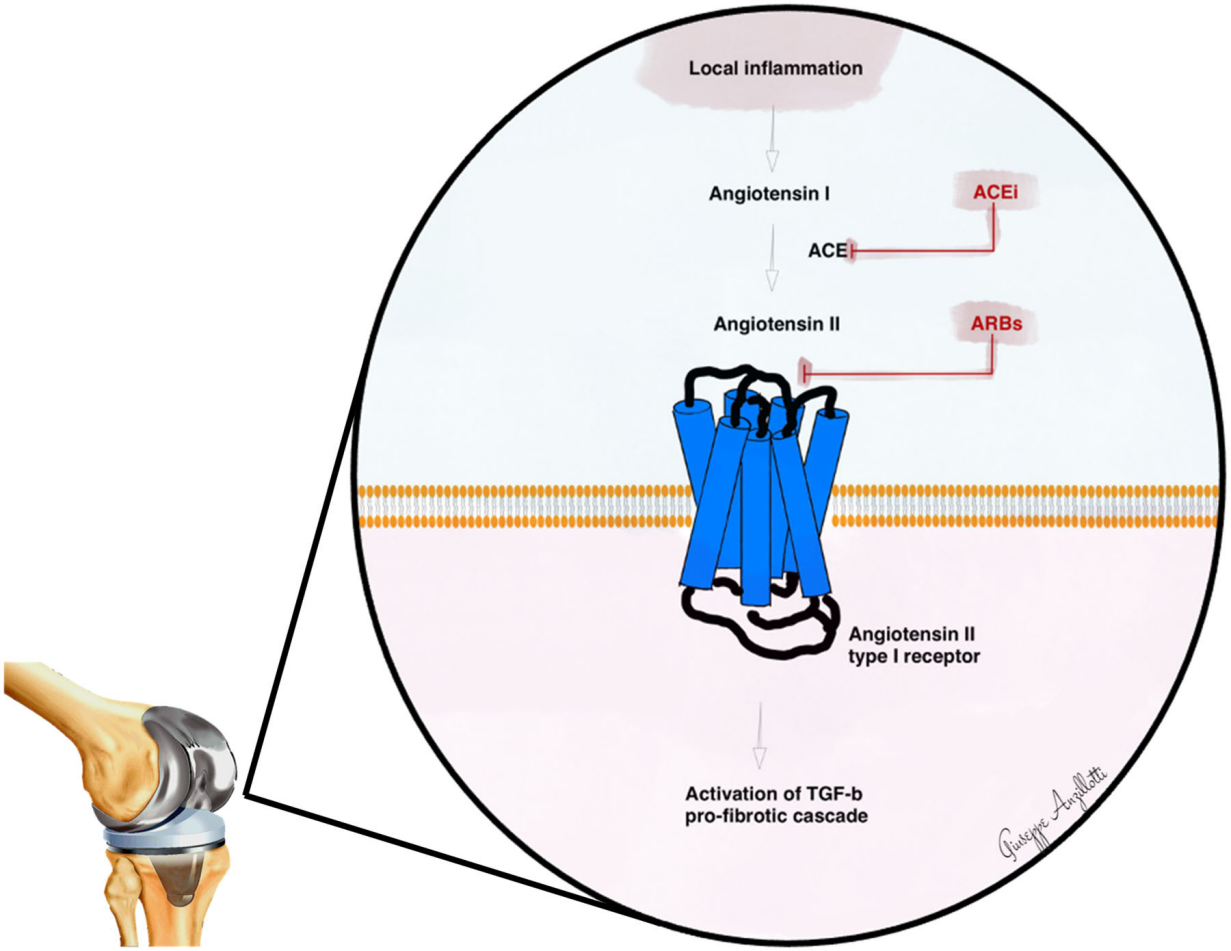
Schematic representation of the role of RAAS pathway in the fibrotic cascade. RAAS, renin–angiotensin–aldosterone system.

Currently, one of the most attractive approaches able to indirectly antagonise the *TGF‐beta 1* is the downregulation of renin–angiotensin–aldosterone system (RAAS), resulted effective and with minimal side effects both in pre‐clinical and clinical studies [5, 50]. This could be achieved by both the inhibition of the angiotensin‐converting‐enzyme which converts Angiotensin I into Angiotensin II (via ACE inhibitors) or through the direct blockage of the Angiotensin II receptor (via Angiotensin receptor blockers) (Figure 1).

Hence, whether the administration of angiotensin receptor blockers (ARBs) or angiotensin‐converting‐enzyme inhibitors (ACEi) might represent a preventive treatment to avoid the onset of arthrofibrosis remains an unsolved conundrum. The aim of the present systematic review is to analyse the available evidence regarding the use of ACEi/ARBs to prevent post‐operative knee arthrofibrosis following TKAs.

## MATERIALS AND METHODS

Extensive research on the PubMed, Cochrane and Google Scholar databases was performed on 8 July 2024, using the following words: (losartan OR angiotensin OR valsartan OR sartan OR eprosartan OR irbesartan OR candesartan OR telmisartan) OR (captopril OR enalapril OR lisinopril OR ramipril OR perindopril OR quinapril OR fosinopril OR benazepril OR angiotensin OR ACE inhibitors OR ACEi) AND (stiffness OR fibrosis OR arthrofibrosis) AND (knee). Screening was conducted by two independent reviewers (AB and GA). Eventual discrepancies were solved after discussion with the senior researcher (BDM). First, the articles were screened by title and abstract. Our systematic research adopted the following inclusion criteria: (1) clinical trials of any level of evidence; (2) written in English; (3) studies conducted on humans; and (4) evaluating the antifibrotic effects of ACE inhibitors or ARBs administered for TKA surgeries. Exclusion criteria were preclinical studies; expert opinions; reviews and trials evaluating the effects of ACE inhibitors or ARBs not related to their antifibrotic effect after TKA procedure.

**Figure 2:**
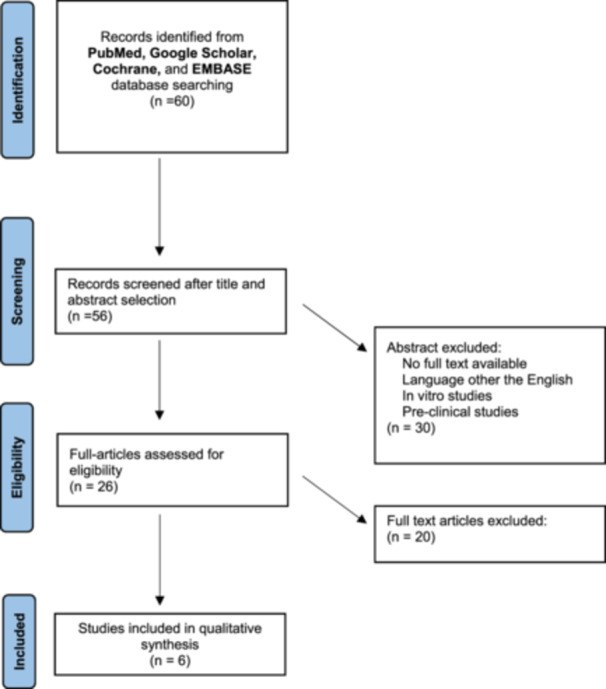
PRISMA (Preferred Reporting Items for Systematic Review and Meta‐Analysis) flowchart of the systematic literature review.

Afterwards, a screening of the full texts of the selected articles was performed. The reference list of all the retrieved articles was further screened for identification of potentially relevant studies. A PRISMA (Preferred Reporting Items for Systematic Review and Meta‐Analysis) flowchart of the systematic literature review is reported in Figure 2. Data were then extracted and collected in a database to be analysed for the purpose of the present review.

Given the retrospective nature of the studies included with no‐randomised design, the risk of bias was assessed with the ROBINS‐I tool [49].

## RESULTS

A total of six studies were ultimately included in the present systematic review. All six studies investigated the use of ARBs and ACEis for the prevention of post‐operative arthrofibrosis in patients undergoing TKA. Four out of six studies focused exclusively on ARBs. A synopsis of the features of the included studies is reported in Table 1.

**Table 1:** Clinical studies regarding the use of ARBs and ACEis for the prevention of post‐operative arthrofibrosis.

Publication	Study design and level of evidence	Therapeutic protocol	Outcome	Patient characteristics	Follow‐up (months)	Main findings
Hernandez et al.	Retrospective, III	Prescription for ARBs before and after surgery	Post‐operative MUA or arthrofibrosis diagnosis in patients undergoing TKA	3‐month cohort: 11,334 treated vs. 123,093 controls Sex: M‐F = – Age: – 12‐month cohort 14,639 treated vs. 84,190 Sex: M‐F = – Age: –	2 cohorts: 3 and 12	Males, obese patients and >55 years had decreased odds of MUA and arthrofibrosis. Unadjusted analyses suggested a statistically significant improvement in arthrofibrosis and MUA rates at 1 year with use of ARBs. No significant difference in the rate of arthrofibrosis or MUA post‐operative in patients treated with ARBs when adjusted for age, sex, obesity and comorbidities.
Arraut et al.	Retrospective, III	Losartan prescription at least 3 months before TKA	Post‐operative ROM, rate of MUA, readmission, reoperation, and revision in patients undergoing TKA	79 pts takers vs. 79 pts non‐takers Sex: M‐F = 38–120 Age: 66.7 years takers vs. 66.43 years non‐takers	3	No significant difference in post‐operative ROM, readmission, rate of MUA, or all‐cause revision in patients treated with losartan
Albright et al.	Retrospective, III	ARB's prescription for at least 3 consecutive months immediately after TKA	Post‐operative MUA, LOA, aseptic loosening, periprosthetic fractures, and revision arthroplasties in patients undergoing TKA	82,065 ARBs takers vs. 1,217,041 ARBs non‐takers Sex M: 29,872 ARBs takers vs. 332,057 ARBs non‐takers Age: 70 years ARBs takers vs. 67 years ARBs non‐takers	24	ARB use was associated with decreased rates of MUA (OR = 0.94, arthroscopy/LOA (OR = 0.86), aseptic loosening (OR = 0.71), periprosthetic fracture (OR = 0.58), and revision (OR = 0.79) at 2‐year follow‐up. The study also found this association to be the primary result of losartan use (OR 1⁄4 0.94), rather than valsartan or olmesartan.
Premkumar et al.	Retrospective, III	Prescription of at least one between ACEi, statins, ARBs, or COX 2 inhibitors, during the 3 months before or after surgery	Post‐operative MUA	24,266 using ACEi/ARBs (ACEi = 16,980, ARBs = 7286) Sex M% = 40.1 Age entire cohort: 57.5 years	3	Perioperative use of any antifibrotic medication—especially ARB—was associated with a lower likelihood of undergoing MUA. When adjusted for age, sex, comorbidities, opioid use, length of stay, perioperative use of specific ACEi (OR 0.91), and ARBs, namely losartan (OR 0.80), remained significantly associated with lower rates of MUA.
Langston et al.	Retrospective, III	Prescription of ACEi or ARBs at the time of surgery	Success or failure of achieving 118° total ROM post‐operatively	141 patients (ACEi = 35 pts, ARBs = 19 pts, controls = 87) Sex: M % (ACEi = 19, ARBs = 6, controls = 27) Age: ACEi = 63.6 ± 7.9, ARBs = 66.4 ± 7.8, controls = 61.5 ± 10.0	6	No significant difference in achieving more than 118° of post‐operative ROM between patients taking either ACEi or ARBs and controls.
Rana et al.	Retrospective, III	Prescription of Losartan from the year before up to 90 days after TKA	Post‐operative revision TKA and MUA within 1 year after surgery or ED visit and readmission from 5 to 90 days post‐operatively	25,786 takers vs. 25,786 non‐takers Sex F: 16,192 takers vs. 16,188 non‐takers Age: 68.5 ± 8.9 years takers vs. 68.5 ± 8.9 years non‐takers	12	Post‐operatively, losartan‐treated TKA patients were 1.18 (OR: 0.85) times less likely to be readmitted within 90 days and 1.15 (OR: 0.87) times less likely to undergo MUA within 1 year.

Abbreviations: ACEi, angiotensin‐converting enzyme inhibitors; AKI, acute kidney injury; ARB, angiotensin receptor blocker; COX 2, cyclooxygenase 2; ED, emergency department; JA, joint arthroplasty; LOA, arthroscopic lysis of adhesions; MACCE, major adverse cardiac, and cerebrovascular event; MUA, manipulation under anaesthesia; OR, odds ratio; RAAS, renin–angiotensin–aldosterone system; ROM, range of motion; TKA, total knee arthroplasty.

### Quality assessment

The average risk of bias, according to the ROBINS‐I tool, was moderate (Table 2) [49]. All studies were retrospective and involved a total of 158,310 patients. Inclusion and exclusion criteria were reported for all. Nonetheless, all records were identified for most authors starting from either national or commercially available databases and using Current Procedural Terminology.

**Table 2:** ROBINS‐I tool for the risk of bias assessment of the non‐randomised controlled trials of the included studies.

Author and citation (year pub)	Baseline confounding	Selection of participants	Classification of intervention	Deviation from intended intervention	Missing data	Measurement of outcomes	Selection of reported results	Overall risk of bias
Hernandez et al. (2020)	Moderate	Low	Serious	Low	Moderate	Moderate	Low	Serious
Arraut et al. (2022)	Moderate	Low	Moderate	Low	Low	Moderate	Low	Moderate
Albright et al. (2023)	Moderate	Low	Serious	Low	Low	Moderate	Low	Moderate
Premkumar et al. (2022)	Moderate	Low	Serious	Low	Low	Moderate	Low	Moderate
Langston et al. (2020)	Moderate	Low	Moderate	Low	Low	Moderate	Low	Moderate
Rana et al. (2024)	Moderate	Low	Moderate	Low	Low	Moderate	Low	Moderate

Two studies had a population size inferior to 5000 [3, 30].

Patients' selection was unclear among the retrieved studies since arthrofibrosis was not defined through objective parameters, except for the study from Langston et al. [30] which used the threshold of 118°.

The mean follow‐up was overall quite short, with just two studies having a mean follow‐up over 12 months [2, 41]. Regarding interventions, there was a lack of uniformity, with two studies [3, 41] focusing on losartan only, a single study [30] focusing on ACEi and ARBs alike, two studies [2, 24] solely focusing on other ARBs, and one focusing on ACEi, statins, ARBs and COX 2 inhibitors [39]. Only three [3, 39, 41] studies focused on the potential role of ARBs and ACEi in preventing arthrofibrosis before the intervention.

Information about intervention was scarce for all authors. No single article specified type, setting, dose, frequency, intensity and/or timing of interventions, and no single author clearly remarked whether all selected patients shared the same prescription.

### Clinical outcomes

All studies but one [30] reported the number of patients who underwent MUA. Post‐operative knee range of motion (ROM) was assessed in only two [3, 30] out of six studies. There were only two studies that reported on other complications that occurred [2, 3]. Overall, three [2, 39, 41] out of six studies showed a statistically significant correlation between the use of ARBs and/or ACEi and a lower likelihood of developing arthrofibrosis. In particular, Albright et al. demonstrated how the use of ARB was associated with decreased rates of MUA, arthroscopic lysis of adhesions (LOA), aseptic loosening, periprosthetic fractures and revision 2 years after TKA [2]. That was the study with the biggest cohort and the longest follow‐up. Rana et al. documented statistically significant results as well: in their work, patients under losartan prescription before TKA had significantly lower 90‐day readmission rates and reduced likelihood of undergoing MUA within 1 year compared to controls [41]. Finally, Premkumar et al. showed how the use of any antifibrotic medication in the peri‐operative period was associated with a lower likelihood of undergoing MUA. However, when adjusted for age, sex, comorbidities, opioid use and length of stay, it emerged that perioperative use of ACEi (odds ratio [OR]: 0.91), and ARBs, specifically losartan (OR: 0.80), was significantly associated with lower rates of MUA [39].

Conversely, Langston et al. reported no significant differences between ACEi/ARBs and placebo in the post‐operative ROM. However, the authors arbitrarily focused on a specific cut‐off for ROM (118°) [30]. Finally, Hernandez et al. showed a statistically significant advantage for ACEi/ARBs users in terms of reduced arthrofibrosis and MUA rates at 1 year, even if no differences were observed when the analysis was adjusted for age, sex, body mass index and comorbidities [24]. Additionally, Arraut et al. reported no significant difference in post‐operative ROM, readmission, rate of MUA or all‐cause revision in patients treated with losartan [3].

### Timing and dosage

Two studies did not specify the drugs used, but indicated only the pharmaceutical class [24, 30]. Four out of six studies focused their results specifically on losartan [2, 3, 39, 41]. Furthermore, Albright et al. even reported benefits primarily associated with losartan use (OR: 0.94), rather than valsartan or olmesartan, from the same class of drugs [2]. Dosage of the drugs was not reported in any of the studies, though generally described within limits for cardiological conditions' treatment. The timing of administration and duration of the treatment was also variable and often imprecisely reported among the studies. Only the study from Albright et al. included patients administered with drugs only after surgery, for at least 3 months [2]. Premkumar et al. indistinctively included patients who took drugs during the 3 months before or after surgery [39]. Hernandez et al. and Rana et al. both focused their work on patients who took the drugs before and after the surgery. In particular, the drugs were administered within the study time‐frame in the former, and from the year before up to 3 months after surgery, in the latter study [24, 41]. Langston et al. referred to patients who were taking those classes of drugs ‘at the time of’ the surgery [30]. Finally, Arraut et al. included patients taking losartan at least 3 months before the surgery [3].

## DISCUSSION

The main finding of the present review is that the inhibition of RAAS could have a potential in stiffness prevention in patients undergoing TKA. However, the heterogeneity of the included studies warrants caution in interpreting the results, given the wide diversity of cohorts, drugs and therapeutical protocols examined by each retrieved study.

We believe that the present review offers a valuable contribution to the discussion on such a challenging clinical condition: representing almost 4% of adverse events after TKAs, idiopathic stiffness ranks for almost 10% among revision causes at 5‐year follow‐up [35, 44, 45, 52]. Hence, various strategies have been adopted to try to overcome this common complication, and as is usually the case, when there are multiple strategies to address one problem, none of them works very well. MUA and revision surgery are usually considered the first therapeutical options, even though they suffer from complications such as fracture, patellar tendon avulsion, wound dehiscence and heterotopic bone formation [3, 33]. Moreover, these types of interventions do not address the biological cause of stiffness, and this is demonstrated by previous clinical studies evidencing that up to one out of four TKA revisions performed for idiopathic stiffness requires a second revision surgery [27]. The major issue in idiopathic stiffness management lies in its multifactorial aetiology, as no single responsible has been found, whereas multiple risk factors such as female sex, young age, smoking, African American race and prior knee surgery have been proposed [34, 37, 57]. On the pathogenetic side, translational studies unveiled the underlying mechanism leading to arthrofibrosis after surgical trauma. In general, any surgical insult activates immune cell stimulation, resulting in a cytokine storm and prolonged release of inflammatory mediators, including transforming growth factor beta‐1 (TGF‐β1), interleukin‐1 beta (IL‐1β), interleukin‐6 (IL‐6), interleukin‐13 (IL‐13) and interleukin‐133 (IL‐133), which are involved in the differentiation of fibroblasts into myofibroblasts in the surgical site [25, 39]. In physiological conditions, the number of myofibroblasts decreases over time but, in some patients, a positive feedback is activated and the *TGF‐β1*‐driven inflammatory cascade further stimulates myofibroblasts of the periarticular tissue into exaggerated deposition of collagen, causing pain and restricted joint motion [23, 55]. Previous studies found that angiotensin II is secreted by local myofibroblasts and macrophages at the injury site and, more interestingly, its receptor (AT1R) is usually more present than normal in those sites [5, 6]. When the receptor is activated, it triggers the *TGF‐β1* secretion, activation and signalling, ultimately leading to fibrosis [16, 56, 58]. Various studies on drugs able to counteract fibrosis have been conducted in the past in other organs, with significant results, whereas studies on musculoskeletal tissue are scarce [11, 26, 46]. Shohat et al. conducted a retrospective study on patients treated with drugs for deep venous thrombosis (DVT) prophylaxis after primary TKA: they concluded that aspirin prophylaxis was associated with lower rates of MUA following TKA compared to warfarin and other DVT chemoprophylactic agents when grouped together [47].

Salmons et al. conducted a study on 12,735 knees on the possible effects of the medication used by patients in the setting of knee arthrofibrosis prevention [43]. Among the drugs analysed, they reported a statistically significant reduced risk of arthrofibrosis after TKA in patients with preoperative use of non‐steroid anti‐inflammatory drugs and patients under oral corticosteroids in the peri‐operative period. Both these drugs helped in reducing the need for MUA for all patients, and both the previous studies evidenced how a potential way to prevent fibrosis is to counteract inflammation.

ACEi/ARBs are drugs commonly used in internal medicine departments to inhibit RAAS, one of the most studied pathways regulating electrolyte balance and blood pressure [14]. However, components of the classic RAAS are present even locally in various tissues throughout the body, including the skin, bone marrow and heart [36, 54]. These local RAASs are able to activate independently from the systemic chain and can lead to both pathological and physiological events [9, 53]. Hence, ACEi/sartans targeting the RAASs may have effects on both levels, and they were found able to inhibit the *TGF‐β*‐driven pro‐fibrotic cascade [5, 18]. Preclinical studies showed encouraging results. Baranowski et al. conducted a randomised blinded experiment on 24 rats undergoing joint injury and immobilisation immediately after surgery [4]. They found that losartan reduced the number of myofibroblasts in the joint capsule and was able to increase the length of the inferior joint capsule compared to placebo. Hashemi et al. resected the anterior cruciate ligament of 24 healthy rabbits to induce arthrofibrosis and osteoarthritis [20]. Their findings evidenced how the administration of captopril for 30 days after the injury was able to reduce the fibrosis of the joint compared to placebo. Kobayashi et al. evaluated the effects of losartan with or without muscle‐derived stem cells (MDSCs) in recovery after induced tibialis anterior muscle injury in 48 mice [28]. The authors found that the combination of losartan and MDSC was the most effective in preventing scar tissue formation and improving recovery after muscle injury. Interestingly, they examined doses from 3 to 300 mg kg^−1^ day^−1^. The dose of 10 mg/kg/day is equivalent to the clinically relevant dose used to treat hypertension in humans, but, at this dosage, it did not accelerate muscle healing thus suggesting that that human‐equivalent doses used for hypertension are not enough to prevent arthrofibrosis after TKA.

Furthermore, Bedair et al. evaluated the effects of two different dosages of losartan on 40 mice with bilateral gastrocnemius lacerations [5]. The authors showed how a dose‐dependent, significant reduction in fibrous tissue formation and muscle regeneration occurred at 3‐week and 5‐week follow‐ups, even showing histological dose‐dependent muscle healing signs.

Despite encouraging preclinical studies, our systematic review does not outline such amazing results in vivo. Moreover, it is important to underline that, despite the inhibition of the RAAS axis is achievable with different classes of molecules, most of the studies retrieved focused specifically on ARBs, in particular losartan, which is the drug on which the evidence of antifibrotic evidence is more robust [7, 8, 10, 12, 17].

In particular, only two [2, 41] out of six studies reported favourable results with the use of ARBs alone and, more interestingly, these are the studies with the longest follow‐up period, suggesting a potential time‐dependent role of these drugs. Nonetheless, the quality and the design of studies prevented any further conclusion in terms of dose and type of ARB used. Moreover, ARBs were also considered effective in the study by Premkumar et al., despite the authors included in their analysis different classes of drugs with a short follow‐up period [39].

Furthermore, none of the included studies discusses the complication rate related to ACEi/ARBs administration. Accordingly, this pharmacological class is associated with potential side effects such as hyperkalemia, hypotension and worsening or renal function as well as dry cough and angioedema in case of ACEi administration [21, 29, 42, 59]. Finally, the current cost‐effective balance seems against their prescription with the purpose of avoiding arthrofibrosis after TKA.

In conclusion, as RAAS inhibition seems promising in terms of anti‐fibrotic effect in pre‐clinical models and has widely demonstrated its efficacy on other organs, future investigations in terms of efficacy, timing and dosage are mandatory to confirm the role of these drugs for this specific clinical indication.

Nonetheless, the present study suffers from some relevant limitations. First of all, the quality of the retrieved evidence is of high concern due to the wide variability in drugs adopted and the lack of prospective studies or RCTs conducted on this topic. In fact, the majority of results are derived from registry studies, which often lack uniformity in treatment regimens given to diverse hospital protocols. Additionally, the absence of detailed information about intervention makes impossible the drawing of a definitive clinical conclusion. Moreover, the patients' selection itself remains unclear. Most of the studies did not specify the criteria used to include patients affected by arthrofibrosis and it is only assumable that they rely on the definition from the Knee Society in 2013, which defined stiffness as a ‘Limited ROM as reported by the patient and demonstrated in a physical examination with extension limited to 15° short of full extension or flexion <90° (not applicable if preoperative arc of motion <75°)’ [22].

Furthermore, although TKA is a well‐standardised procedure, the influence played by different intra‐operative choices (e.g., surgical exposure, eventual resurfacing of the patella, alignment techniques and use of toxic drugs), and different post‐operative analgesia and rehabilitation protocols might play a significant role in causing the onset of post‐operative arthrofibrosis.

## CONCLUSIONS

The RAAS antagonism could have a potential in stiffness prevention in patients undergoing TKA. However, the heterogeneity of the included studies warrants caution in interpreting the results, given the wide diversity of cohorts, drugs and therapeutical protocols examined by each retrieved study. Hence, given the side effects and the limited evidence available, the use of ACEi/sartans for the sole purpose of avoiding arthrofibrosis after TKA is not currently recommended.

## AUTHOR CONTRIBUTIONS

Giuseppe Anzillotti, Pietro Conte, Paolo Queirazza and Alberto Bulgarelli wrote the draft of the paper. Screening was conducted by two independent reviewers (Alberto Bulgarelli and Giuseppe Anzillotti). Eventual discrepancies were solved after discussion with senior researchers (Berardo Di Matteo). Andreas H. Gomoll, Berardo Di Matteo and Elizaveta Kon contributed to the conception of the manuscript and critically revised the draft. All authors read, revised and approved the final manuscript.

## CONFLICT OF INTEREST STATEMENT

The authors declare no conflict of interest.

## ETHICS STATEMENT

The ethics statement is not available.

## Data Availability

All retrieved data were included in the present manuscript.
